# Involvement of von Willebrand factor and botrocetin in the thrombocytopenia induced by *Bothrops jararaca* snake venom

**DOI:** 10.1371/journal.pntd.0009715

**Published:** 2021-09-03

**Authors:** Camila Martos Thomazini, Ana Teresa Azevedo Sachetto, Cynthia Zaccanini de Albuquerque, Vânia Gomes de Moura Mattaraia, Ana Karina de Oliveira, Solange Maria de Toledo Serrano, Ivo Lebrun, Katia Cristina Barbaro, Marcelo Larami Santoro

**Affiliations:** 1 Laboratório de Fisiopatologia, Instituto Butantan, São Paulo-SP, Brazil; 2 Programa de Pós-Graduação em Clínica Médica, Faculdade de Medicina, Universidade de São Paulo, Sao Paulo-SP, Brazil; 3 Biotério Central, Instituto Butantan, São Paulo-SP, Brazil; 4 Laboratório de Toxinologia Aplicada, Center of Toxins, Immune-Response and Cell Signaling (CeTICS), Instituto Butantan, São Paulo-SP, Brazil; 5 Laboratório de Bioquímica e Biofísica, Instituto Butantan, São Paulo-SP, Brazil; 6 Laboratório de Imunopatologia, Instituto Butantan, São Paulo-SP, Brazil; Monash University, AUSTRALIA

## Abstract

Patients bitten by snakes consistently manifest a bleeding tendency, in which thrombocytopenia, consumption coagulopathy, mucous bleeding, and, more rarely, thrombotic microangiopathy, are observed. Von Willebrand factor (VWF) is required for primary hemostasis, and some venom proteins, such as botrocetin (a C-type lectin-like protein) and snake venom metalloproteinases (SVMP), disturb the normal interaction between platelets and VWF, possibly contributing to snakebite-induced bleedings. To understand the relationship among plasma VWF, platelets, botrocetin and SVMP from *Bothrops jararaca* snake venom (BjV) in the development of thrombocytopenia, we used (a) Wistar rats injected s.c. with BjV preincubated with anti-botrocetin antibodies (ABA) and/or Na_2_-EDTA (a SVMP inhibitor), and (b) VWF knockout mice (*Vwf*^*-/-*^) injected with BjV. Under all conditions, BjV induced a rapid and intense thrombocytopenia. In rats, BjV alone reduced the levels of VWF:Ag, VWF:CB, high molecular weight multimers of VWF, ADAMTS13 activity, and factor VIII. Moreover, VWF:Ag levels in rats that received BjV preincubated with Na_2_-EDTA and/or ABA tended to recover faster. In mice, BjV caused thrombocytopenia in both *Vwf*^*-/-*^ and C57BL/6 (background control) strains, and VWF:Ag levels tended to decrease in C57BL/6, demonstrating that thrombocytopenia was independent of the presence of plasma VWF. These findings showed that botrocetin present in BjV failed to affect the extent or the time course of thrombocytopenia induced by envenomation, but it contributed to decrease the levels and function of plasma VWF. Thus, VWF alterations during *B*. *jararaca* envenomation are an ancillary event, and not the main mechanism leading to decreased platelet counts.

## Author summary

Envenomation by snakebites is a major burden to tropical and subtropical areas in the world. Many snake species produce venoms that, when injected into victims, cause bleedings and other associated symptoms and signs. This work aimed to understand the mechanisms that lead to a fall in blood platelet counts after bites by a snake that inhabits in southeastern Brazil, the lance-headed snake *Bothrops jararaca* (popularly known as jararaca). We used experimental approaches to understand the involvement of a protein from jararaca venom, called botrocetin, and a protein present in our blood (von Willebrand factor) in the fall of platelet counts. We observed that botrocetin alters von Willebrand factor, but this mechanism in not important for the decrease in platelet counts. We show that jararaca snake venom disturb blood platelets in a complex and intricate way, and that other venom compounds are involved in the decrease of platelet counts during snakebite envenomation.

## Introduction

Among the Neglected Tropical Diseases categorized by the World Health Organization (WHO), snake envenomation has high incidence and severity, especially in tropical and developing countries [1]. *Bothrops* sp snakes inhabit Central and South America and represent the main agent of snakebites in Brazil. The species *Bothrops jararaca* is of great epidemiological importance in southeastern Brazil [2]. *Bothrops jararaca* snake venom (BjV) is composed by a plethora of toxins that, acting in concert, disrupt the steady state of various physiological systems, including hemostasis. Bites by this snake engender local and systemic manifestations, which may result in clinical complications, e.g. kidney failure, shock, hemorrhage or even death. At the site of the bite, edema, bleeding from the fang marks, petechiae, ecchymosis, blisters and necrosis are observed. Systemically, patients frequently manifest hemorrhagic disorders, such as gingival bleeding, epistaxis, suffusions, petechiae and hematuria [3,4]. At the laboratory level, patients present hemostatic alterations–e.g. consumption of blood coagulation factors, particularly of fibrinogen, factor V and VIII, thrombocytopenia, and increased levels of fibrinogen/fibrin degradation products–and inflammatory changes, as increased levels of C-reactive protein and interleukin 6 [4–9]. *In vitro*, BjV induces platelet aggregation in washed platelets, activates blood coagulation and promotes fibrin(ogen)olysis [10–13]. The association between hypofibrinogenemia and thrombocytopenia contributes to the increased incidence of bleeding in patients bitten by *B*. *jararaca* [14], but the development of systemic bleeding also depends on the reduction of platelet function and other alterations in blood coagulation, fibrinolysis, and endothelial cells [15–17].

Thrombocytopenia is consistently observed in envenomation by Viperidae snakes, and it is already known that the magnitude of thrombocytopenia depends on the severity of *B*. *jararaca* envenomation [4]. Interestingly, platelet counts initiate to recover soon after administration of antivenom, as early as 6 h [4], and stimulation of production of new platelets, evidenced by an increase in reticulated platelets during the first 48 h of envenomation [17], also occurs. Taken together, these findings demonstrate that some venom component is directly involved with thrombocytopenia development and that neutralization by antibodies interrupts the process of platelet consumption.

Platelet plug formation is dependent on von Willebrand factor (VWF) function. VWF plays an essential role in primary hemostasis, bridging the binding of platelets to the subendothelium, as well as to other platelets, thereby promoting the formation of the platelet plug in injured vessels. More recently, VWF has also been reported to be involved in other inflammatory and infectious conditions [18]. VWF is a large multimeric glycoprotein (GP) (500 to >10,000 kDa), formed by subunits (approximately 240 kDa) covalently linked by disulfide bonds. Within each VWF subunit, structural domains that bind to collagen, platelet membrane receptors (GPIbα and GPIIb/IIIa), and blood coagulation factor VIII are present; in fact, VWF is the carrier protein of factor VIII in circulation, thus protecting it from clearance [19,20]. VWF is synthesized by endothelial cells and megakaryocytes, and stored in specialized granules of endothelial cells and platelets, under heterogeneous multimeric sizes, including in the form of high molecular weight multimers (HMWM). Constitutive secretion by endothelial cells is responsible for most of the VWF in circulation, but when endothelial cells and platelets are stimulated by inflammatory cytokines or noxious stimuli, additional VWF is released in the bloodstream. Under conditions of elevated shear stress, observed in normal vessels in circulation, globular HMWM-VWF unfolds and is cleaved by ADAMTS13, the metalloproteinase that cleaves VWF specifically at the A2 domain (Tyr1605-Met1606), generating VWF multimers of different molecular masses [18,21–23]. Thus, ADAMTS13 modulates this characteristic heterogeneity in VWF size, which is essential to control the physiological function of VWF, inasmuch as large VWF multimers have a greater potential to induce platelet activation and aggregation than small ones [24]. Thus, for the evaluation of VWF, quantitative and qualitative laboratory assays are required [25]. Von Willebrand disease (VWD) is a disorder caused by quantitative or qualitative defects of VWF, and it can be congenital or acquired. Congenital VWD is one the most prevalent hemostatic disorder in humans, and is characterized by the manifestation of bruising (petechiae, hematomas, and suffusions), prolonged bleeding from minor skin trauma, and prolonged bleeding from mucosal surfaces. Acquired VWD occurs when acquired conditions–particularly associated with neoplastic, immune, infectious or cardiovascular disorders–cause functional impairment or reduce plasma levels of VWF, interfering in hemostasis [26–28].

BjV contains three main toxin families: snake venom metalloproteinases (SVMP, 33.6%), snake venom serine proteases (SVSP, 22.8%), and C-type lectin/lectin-like proteins (18.2%) [29]. SVMP play a significant role in the pathogenesis of envenomation, and are considered primary factors responsible for hemorrhage. They have a wide range of activities, including activation of blood coagulation prothrombin and factor X, and proteolysis of collagen, basement membrane components, fibrinogen/fibrin, and VWF [13,14,30–33]. The second class of toxins most abundant in BjV is SVSP, and it is known that they mainly affect blood coagulation factors, fibrinolysis, protein C, kininogen, and blood platelets [34]. C-type lectin-like proteins (CTLP) are a family of non-enzymatic proteins, abundantly present in *Bothrops* venoms, which may profoundly alter the function of blood platelets, blood coagulation, endothelial cells and immune cells. Their basic structures consist of heterodimers, formed from α and β subunits or chains, whose molecular masses are around 14–16 kDa and 13–15 kDa, respectively, covalently linked by disulfide bridges. They have domains related to carbohydrate recognition, but they may bind to their target proteins independently of carbohydrates or calcium [35–39]. CTLP are plastic molecules, and hitherto it is not possible to predict if they act as agonists or antagonists based on their amino acid sequence [37]. Five CTLP have been isolated and characterized in BjV that alter either blood coagulation (jararaca IX/X-binding protein and bothrojaracin) or blood platelets (botrocetin, botrocetin-2, and jararaca GPIbα-binding protein (GPIb-BP)) [40–46].

Interestingly, clinical manifestations of hemostatic disorders in *B*. *jararaca* snakebite envenomation call to mind clinical manifestations of VWD. Levels of circulating VWF markedly vary in patients bitten by *B*. *jararaca* [14], but a reduction in HMWM-VWF was noticed under more controlled experimental conditions [47]. In addition, SVMP that cleave VWF share structural domains with those of ADAMTS13, including the metalloproteinase domain, followed by a disintegrin-like and cysteine-rich domains [48]. For example, jararhagin, a SVMP isolated from *B*. *jararaca*, cleaves VWF at three Gly–Leu bonds, at positions 1242–1243, 1674–1675 and 1922–1923 [31]. However, besides enzymes that cleave VWF, three CTLP from BjV–botrocetin, botrocetin-2 and jararaca GPIbα-binding protein (GPIb-BP)–have been characterized and shown to interact simultaneously with VWF and platelets. Botrocetin was isolated in 1978 [49] from BjV, and its structure and mechanism of action have been well characterized. It is a heterodimer protein that binds to both the A1 domain of VWF and platelet GPIba, without inducing any conformation change in neither of them, thus acting as a biological brace [50]. Purified 2-chain botrocetin causes the rapid consumption of circulating platelets and plasma VWF when injected i.v. in high doses in normal rats, pigs and dogs, but is innocuous to induce thrombocytopenia in VWD animals, showing that the thrombocytopenia was dependent on basal VWF levels in circulation [51–53]. Botrocetin-2, which was cloned from *B*. *jararaca* venom gland, shows a high similarity to primary botrocetin in regard to its structure, immunogenicity and platelet agglutinating activity [41]. On the other hand, GPIb-BP binds to platelet GPIbα, but does not induce platelet activation, and, in fact, it thwarts VWF to bind GPIbα and inhibits botrocetin and ristocetin-induced platelet aggregation [43,54]. GPIb-BP has a high similarity to botrocetin, and was reported to induce thrombocytopenia in mice and guinea pigs [42]. Interestingly, despite the high amino acid similarity between botrocetin, bothrojaracin, and jararaca IX/X binding protein found in BjV, polyclonal antibodies anti-native bothrojaracin, a CTLP that inhibits thrombin, shows low cross-reactivity with native botrocetin and jararaca IX/X binding protein, indicating a higher degree of selectivity of antibodies [55].

By investigating the mechanisms and toxins involved in the coagulopathy and thrombocytopenia in individuals envenomed by *B*. *jararaca* snakes, we observed that thrombocytopenia and consumption of coagulation factors are independent events in envenomation, and that thrombocytopenia is independent of the action of SVMP and/or SVSP [56,57]. Indeed, our results implied that other venom toxin families might be involved in engendering thrombocytopenia. Once purified botrocetin induces thrombocytopenia by a mechanism that is dependent on VWF, we investigated herein, using two experimental models, whether botrocetin and VWF are involved in BjV-induced thrombocytopenia, as the comprehension of these pathophysiological events may lead to better treatments of hemostatic disorders of patients bitten by *B*. *jararaca* snakes.

## Results

### BjV-induced decrease in fibrinogen and factor V levels is blocked by Na_2_-EDTA

All rats injected with BjV had high levels of circulating venom throughout 24 h, but venenemia tended to peak at 6 h and to decrease at 24 h. No statistically significant difference was noticed between BjV preincutated with saline or any treatment used, demonstrating that the entrance of venom proteins in circulation was not hindered (Fig A in S1 Text).

**Fig 1:**
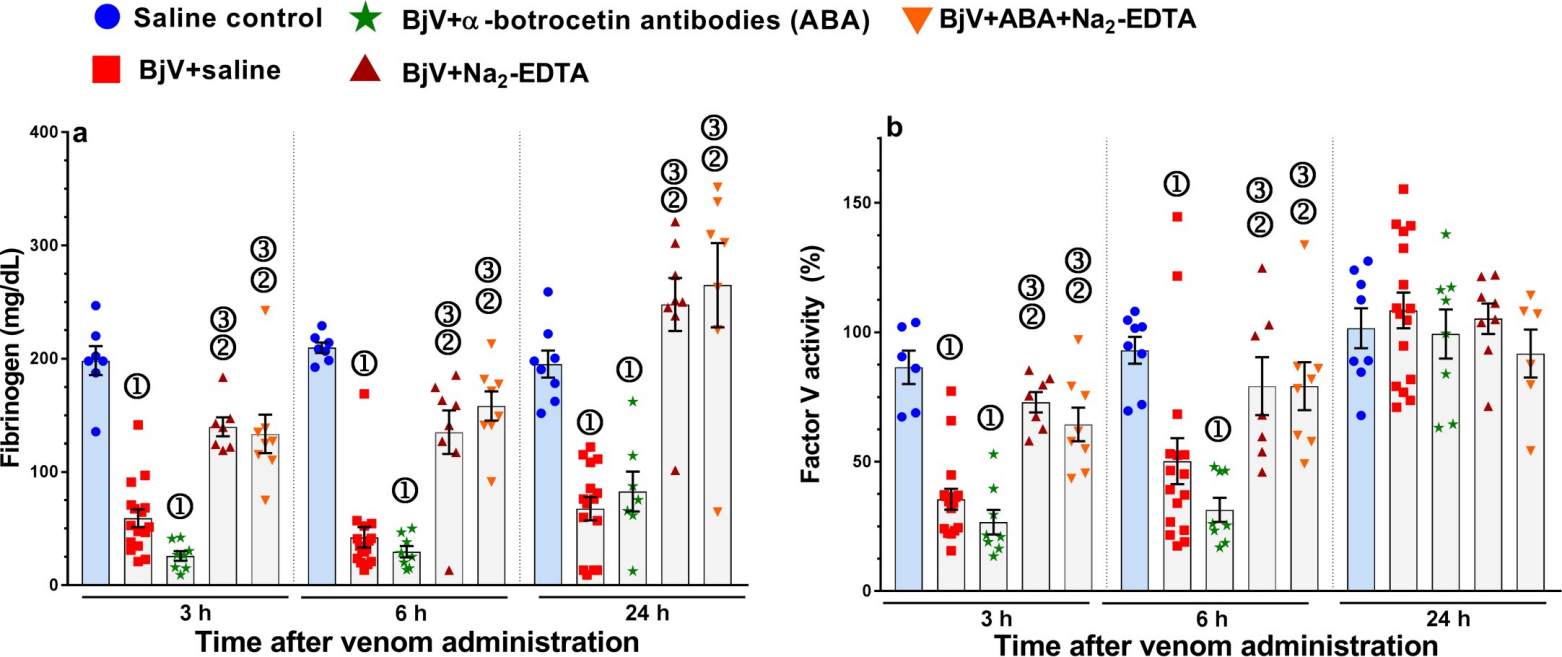
Plasma fibrinogen (**a**) and factor V (**b**) levels in rats 3, 6 and 24 h after BjV administration. Rats were injected with saline alone (saline control, blue bars), or BjV previously incubated with saline (BjV+saline), anti-botrocetin antibodies (ABA), BjV+Na_2_-EDTA, or BjV+ABA+Na_2_-EDTA. ①—Group statistically different (p <0.05) from the saline control group at the respective time. ②- Group statistically different (p <0.05) from the BjV+saline group at the respective time. ③—Group statistically different (p <0.05) from the BjV+ABA group at the respective time. Data are expressed as mean ± s.e.m (n = 6–16 rats/group per time period).

As expected [56,57], rats injected with BjV showed marked hypofibrinogenemia, which initiated to recover only at 24 h (Fig 1). The falls in fibrinogen and factor V levels were completely blocked by Na_2_-EDTA alone or in combination with ABA, demonstrating that SVMP are essential for consumption coagulopathy in rats [57].

### Thrombocytopenia induced by crude BjV is not caused by botrocetin

**Fig 2:**
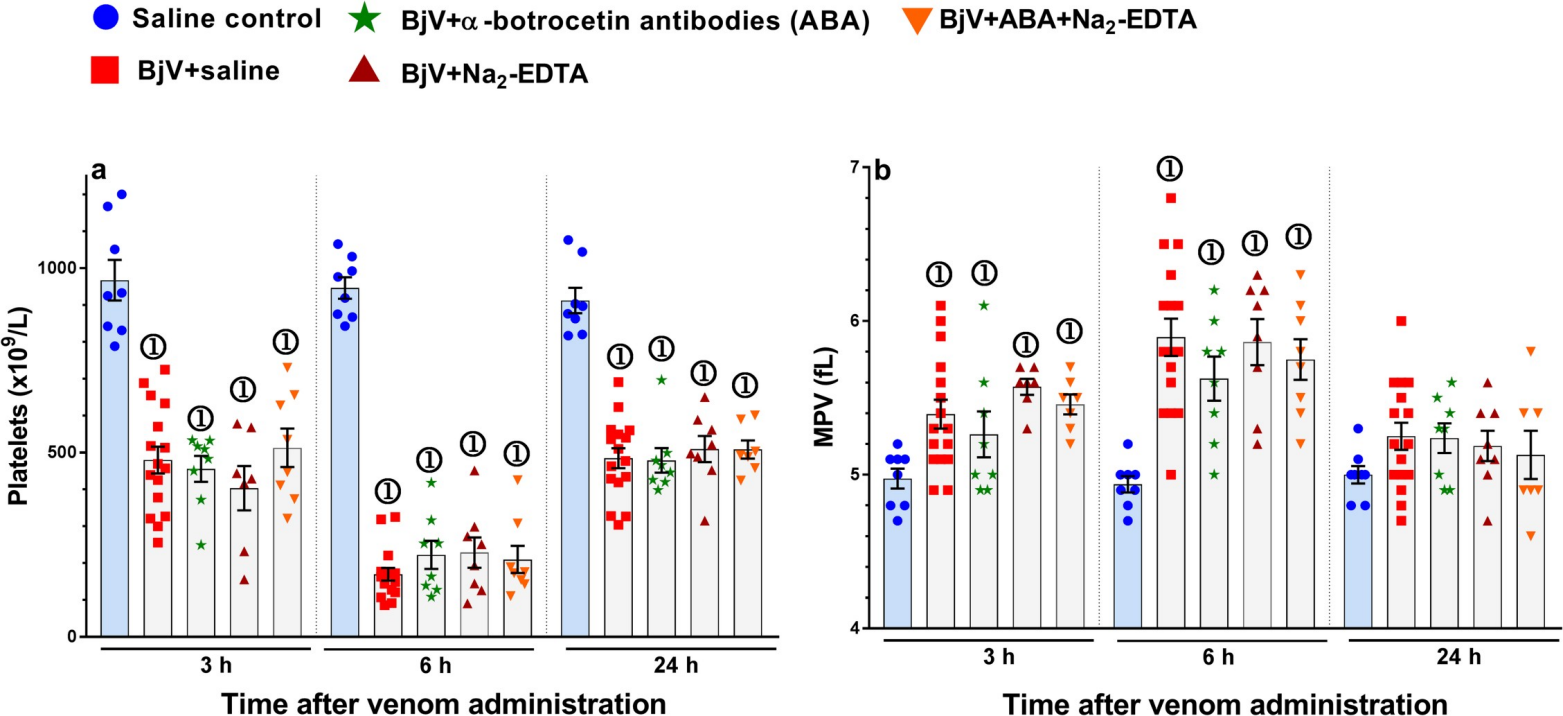
Platelet counts (**a**) and mean platelet volumes (MPV, **b**) in rats 3, 6 and 24 h after BjV administration. Rats were injected with saline alone (saline control, blue bars), or BjV previously incubated with saline (BjV+saline), anti-botrocetin antibodies (ABA), BjV+Na_2_-EDTA, or BjV+ABA+Na_2_-EDTA. ①—Group statistically different (p <0.05) from the saline control group at the respective time. Data are expressed as mean ± s.e.m (n = 6–16 rats/group per time period).

Thrombocytopenia is a frequent finding during *B*. *jararaca* envenomation [4,16,57,58]. In rats, the nadir of platelet counts occurred at 6 h, which could be associated with higher levels of BjV in bloodstream, while restoration of normal platelet counts initiated to occur at 24 h (Fig 2A). In concert with these findings, higher values of mean platelet volume (MPV) were observed in all groups injected with BjV at 6 h, suggesting that young reticulated platelets were being shed from megakaryocytes, as a compensatory mechanism for platelet consumption [8] (Fig 2B).

Thrombocytopenia occurred in all rats injected with BjV, and preincubation of BjV with ABA or Na_2_-EDTA could not prevent it (Fig 2A). ABA could not even partially mitigate the fall in platelet counts, evidencing that botrocetin was not the main toxin involved in the thrombocytopenia caused by BjV. Although botrocetin has platelet agglutinating activity *in vitro*, and causes thrombocytopenia when administered i.v. in high doses to rats [10], its inhibition by preincubation of BjV with ABA evidenced that botrocetin was not relevant for triggering thrombocytopenia in rats. Thus, botrocetin exerted a feeble role in evoking thrombocytopenia during envenomation, and other proteins and mechanisms may be attributed to it. Moreover, the present findings (Fig 2) confirmed that SVMP had no involvement in venom-induced thrombocytopenia [7,57].

### VWF antigen levels and activity tend to decrease during BjV envenomation

**Fig 3:**
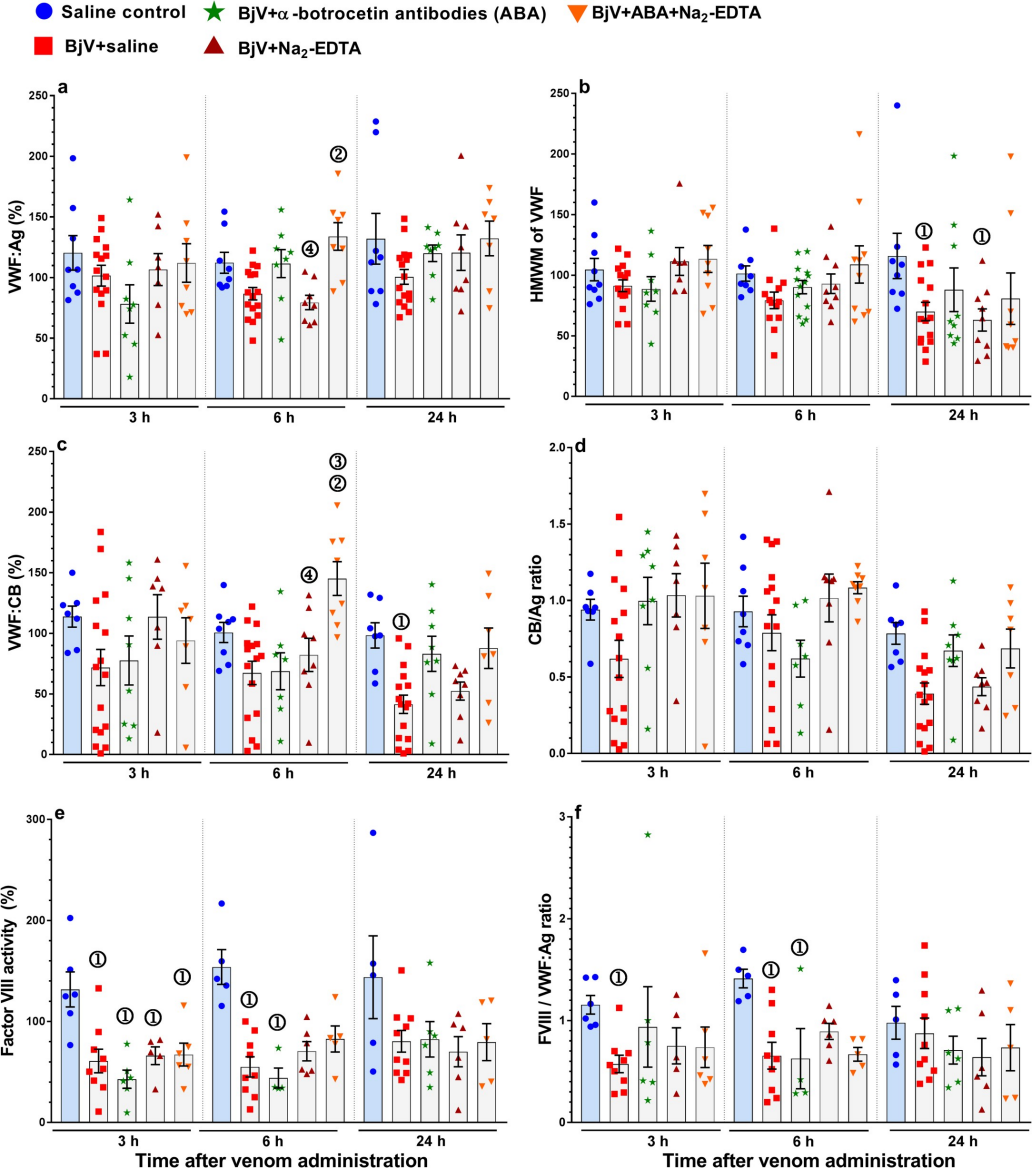
Circulating levels of parameters of VWF and factor VIII function in rats 3, 6 and 24 h after BjV administration. VWF antigen (VWF:Ag, **a**), high molecular weight multimers of VWF (HMWM of VSF, **b**), collagen-binding activity (VWF:CB, **c**), the VWF:Ag/VWF:CB ratio (**d**), factor VIII activity (**e**), and the factor VVIII/VWF:Ag ratio (FVIII/VWF:Ag ratio, **f**). Rats were injected with saline alone (saline control, blue bars), or BjV previously incubated with saline (BjV+saline), anti-botrocetin antibodies (ABA), BjV+Na_2_-EDTA, or BjV+ABA+Na_2_-EDTA. ①—Group statistically different (p <0.05) from the saline control group at the respective time. ②- Group statistically different (p <0.05) from the BjV+saline group at the respective time. ③—Group statistically different (p <0.05) from the BjV+ABA group at the respective time. ④—Group statistically different (p <0.05) from the BjV+ABA+Na_2_-EDTA group at the respective time. Data are expressed as mean ± s.e.m (n = 6–16 rats/group per time period).

Another facet of botrocetin activity is to promptly induce VWF consumption, particularly of HWMW-VWF, when injected i.v. [52,53]. To evaluate the role of plasma VWF in the hemostatic disorder evoked by *B*. *jararaca* envenomation and the participation of botrocetin therein, we determined VWF antigen levels, VWF multimeric distribution (by discontinuous SDS-agarose electrophoresis) and VWF collagen-binding activity in plasma. By analyzing the set of graphs depicted in Fig 3, the first general conclusion drawn from the results is that VWF:Ag, VWF:CB, and HMWM-VWF varies extremely during BjV envenomation (BjV+saline group). Such variation had also been noticed in patients bitten by *B*. *jararaca* [14]. However, there was a general tendency to decrease the levels of VWF:Ag, VWF:CB, and HMWM-VWF, FVIII, CB/Ag ratio and FVIII/VWF ratio in rats from the BjV+saline group in comparison with the saline group (p <0.049), especially at 6 and 24 h (Fig 3). Particularly, preincubation of BjV with ABA and/or Na_2_EDTA tended to prevent the falls in the levels of VWF:Ag, VWB:CB levels, and HMWM-VWF induced by BjV+saline (p = 0.012), indicating that both botrocetin and SVMP were involved in the dynamics of VWF alterations during BjV envenomation.

Although botrocetin promptly reduced plasma VWF levels and platelet counts when administered i.v. in high concentrations [10,11], and that *B*. *jararaca* SVMP may promote proteolysis of VWF *in vitro* [12], a sharp drop in VWF levels, comparable to those of fibrinogen or platelet counts, was not observed in rats from the BjV+saline group. As a mild reduction in VWF:Ag levels was present at 3 and 6 h in the BjV+saline group, we inferred that VWF:Ag was being cleaved and/or consumed, but it was also simultaneously being secreted by stimulation of the endothelium or activated platelets. Although a large variation in the values of HMWM-VWF, determined by discontinuous SDS-agarose electrophoresis, was also observed, there was a clear decrease in the BjV+saline group, particularly at 6 and 24 h (Fig 3B). This change was also observed in mice and rats, as we noticed previously [47].

VWF:CB activity identifies the functional ability of VWF to bind subendothelial collagen, as the larger the size of the multimers, the greater the availability of binding sites for collagen [59]. In fact, a statistically significant correlation was noticed between HMWM-VWF and VWF:CB in our results in the groups BjV+saline and the negative control group from 3 to 24 h (Fig B in S1 Text). Fig 3C shows that despite the high variation in VWF:CB values, there was also a tendency of the mean values of VWF:CB to decrease in the group BjV+saline in comparison with the saline group (p <0.05 at 24 h). Preincubations of BjV with Na_2_-EDTA and/or ABA alleviated the fall in VWF:CB in different extensions depending on the time interval. In the group BjV+saline, the CB/Ag ratio (Fig 3D) also tended to decrease in comparison with the control group, and the lowest values were observed at 24 h. Preincubation of BjV with ABA, alone or combined with Na_2_-EDTA, minimized the alterations in VWF induced by BjV alone, suggesting that botrocetin and SVMP not only influenced the pattern of multimeric distribution, but also the function of VWF to bind to collagen.

### The decrease in factor VIII levels in circulation is independent of VWF alterations

Factor VIII is carried and transported in circulation by VWF [60]. The reduction in factor VIII levels occurred in all groups that received BjV at 3, 6 and 24 h, and differently from VWF, the preincubation of BjV with ABA and/or Na_2_-EDTA did not decrease its consumption (Fig 3E). Rats from the BjV+saline showed a more pronounced reduction in the FVIII/VWF:Ag ratio at 3 and 6 h compared to the saline control (Fig 3F). These findings suggested that the fall in factor VIII is independent of the alterations in VWF during *B*. *jararaca* envenomation, and are caused by other mechanisms.

### ADAMTS13 levels are diminished during envenomation

**Fig 4:**
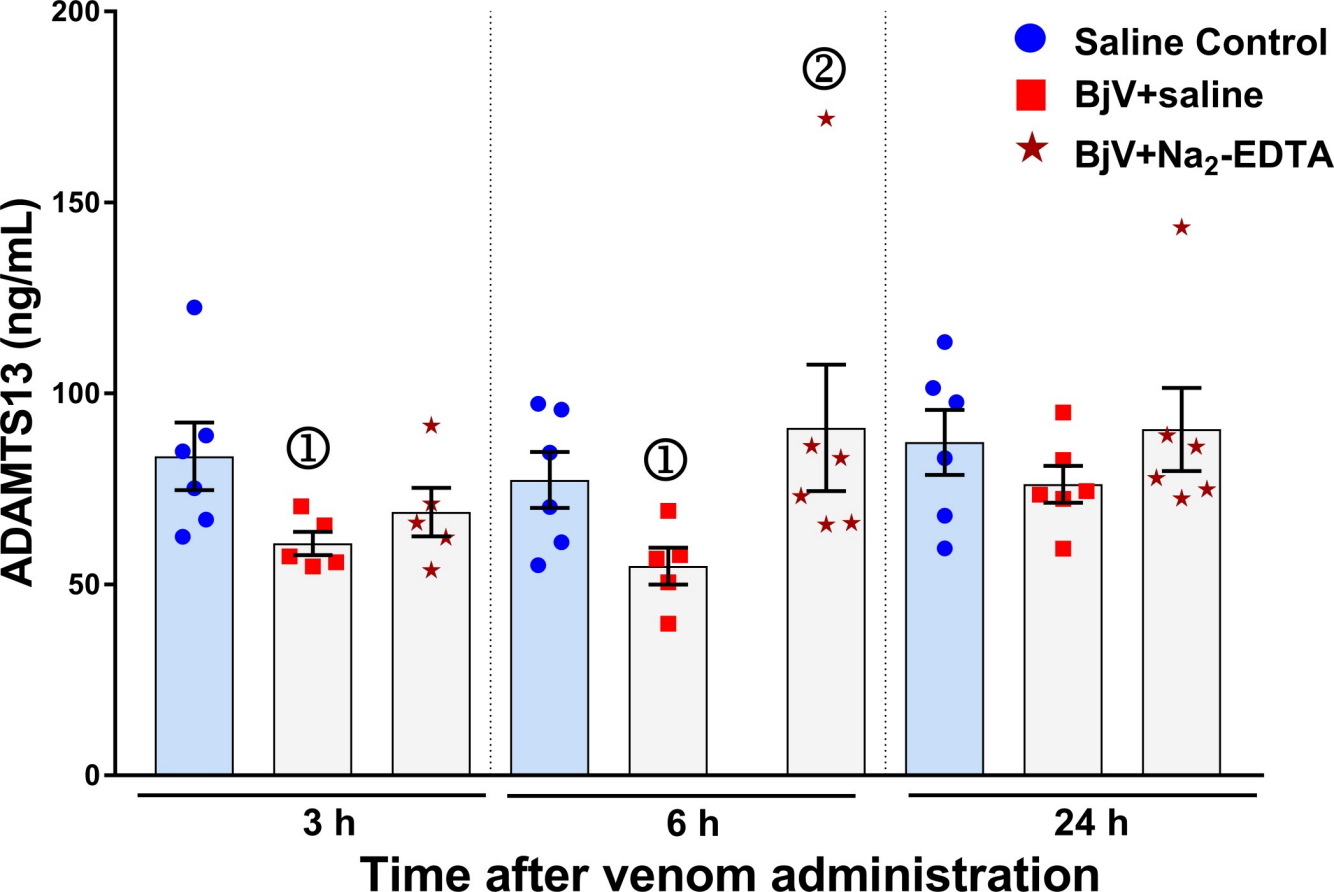
Plasma levels of ADAMTS13 in rats 3, 6 and 24 h after BjV administration. Rats were injected with saline alone (saline control, blue bars), or BjV previously incubated with saline (BjV+saline), and BjV+Na_2_-EDTA. ①—Group statistically different (p <0.05) from the saline control group at the respective time. ②- Group statistically different (p <0.05) from the BjV+saline group at the respective time. Data are expressed as mean ± s.e.m (n = 6–16 rats/group per time period).

Once VWF was proteolyzed in circulation, we also investigated the levels of ADAMTS13, the enzyme that physiologically cleaves VWF [61], using an activity assay that can recognize rat ADAMTS13. During *B*. *jararaca* envenomation, rats had a reduction in ADAMTS13 activity levels (Fig 4). Although there was also a mild decrease in ADAMTS13 levels in the BjV+Na_2_-EDTA group at 3 h, their mean values were slightly higher than those from the BjV+saline. However, at 6 h, there was a significant difference between the groups that received BjV preincubated with saline and Na_2_-EDTA. Thus, the results indicate that ADAMTS13 activity is mildly decreased during envenomation, and that SVMP contribute to diminish ADAMTS13 levels in plasma.

Altogether, these results showed that botrocetin had no participation in the development of thrombocytopenia, and that botrocetin and SVMP had a more evident role in the consumption and cleavage of VWF. In order to double check the findings showing that thrombocytopenia was independent of VWF consumption and botrocetin participation, we used a mouse model deficient in VWF (*Vwf*^-/-^ knockout mice) [62].

### The same magnitude of BjV-induced thrombocytopenia is noticed in C57BL/6 and *Vwf*^*-/-*^ mice

*Vwf*^*-/-*^ and C57BL/6 (background control) mice received the same dose of BjV, and both strains had the same levels of venom in circulation over time (p = 0.951, Fig C in S1 Text). The intensity of local hemorrhage, at the site of BjV injection, was not apparently different from C57BL/6 mice, but the usual fall noticed in RBC counts, hemoglobin and hematocrit (Fig C in S1 Text) at 24 h in BjV-envenomed mice [58] was more intense in *Vwf*^*-/-*^ mice, showing that VWF is involved in bleeding prevention during envenomation. C57BL/6 mice showed increased WBC counts (Fig C in S1 Text) at 24 h, which could be a response to the inflammatory process triggered by envenomation. The same extent of increase in WBC count was not found in *Vwf* ^-/-^ mice, which may be likely due to the deficiency in pro-inflammatory proteins in these mice [63,64].

**Fig 5:**
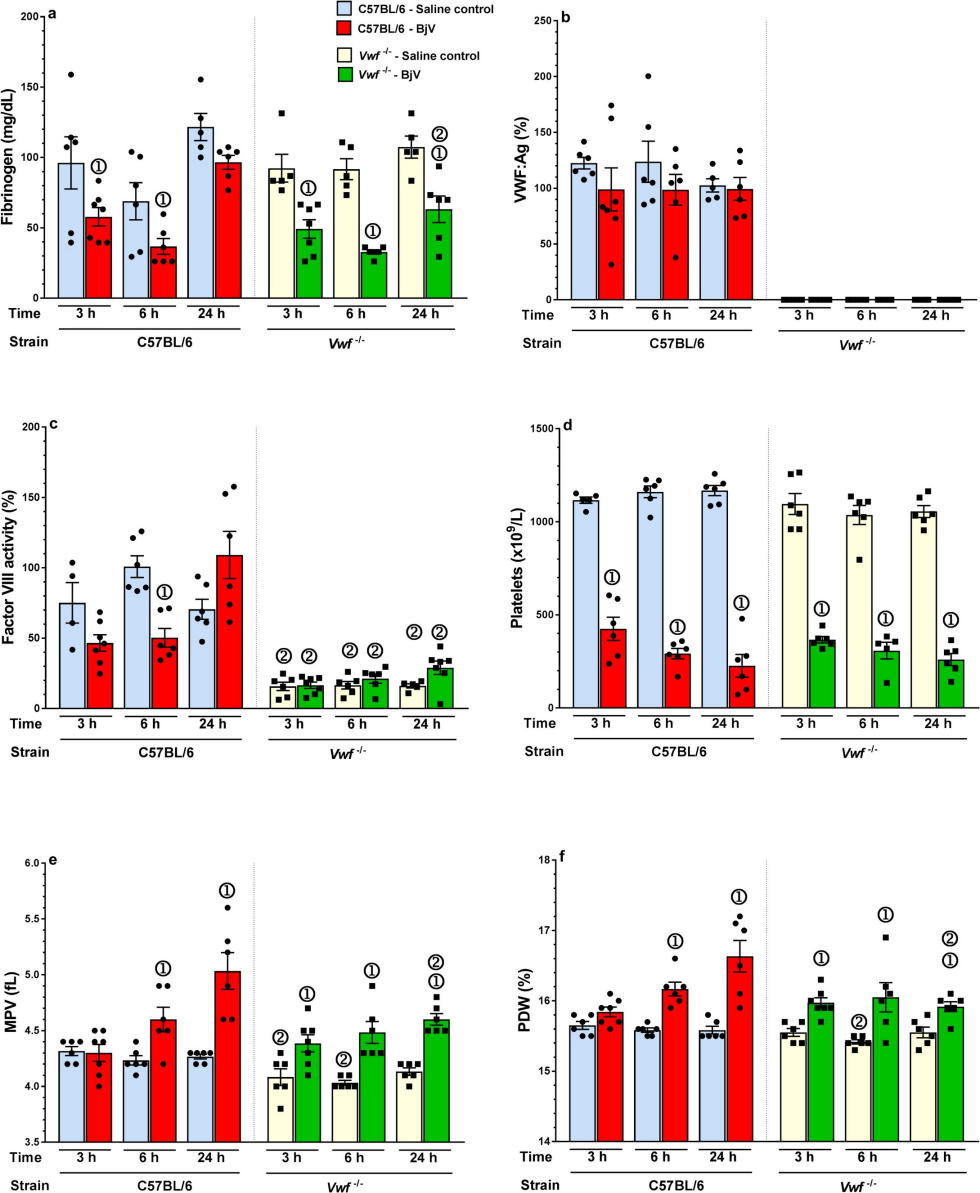
Fibrinogen (**a**), VWF:Ag (**b**), factor VIII activity (**c**), platelets (**d**), MPV (**e**), and platelet distribution width (PDW, **f**) in *Vwf*^*-/-*^ and C57BL/6 mice at 3, 6 and 24 h after injection of saline or BjV. ① Group statistically different (p <0.05) from the saline control group at the respective time. ②- The response of *Vwf*^-/-^ mice was statistically different (p <0.05) from that of C57BL/6 mice at the respective time and treatment. Data are expressed as mean ± s.e.m (n = 5–7 mice/group/time period).

The expected reduction in fibrinogen levels occurred in the BjV+saline group at 3 and 6 h [58] in both strains (Fig 5A). C57BL/6 mice manifested a characteristic recovery in fibrinogen levels at 24 h [58], but it was less pronounced in *Vwf*^*-/-*^ mice (p<0.05). In fact, *Vwf*^*-/-*^ mice have been associated with deficiencies in pro-inflammatory proteins [63,64], which could explain the low recovery of fibrinogen levels [65].

The levels of VWF:Ag in circulation, as expected, could not be detected in *Vwf*^*-/-*^ (Fig 5B). Like rats, plasma VWF levels tended to decrease mildly in C57BL/6 mice injected with BjV, but no statistically significant difference was noticed (Fig 5B). Due to the expected absence of circulating VWF in *Vwf*^*-/-*^ mice [62], the levels of factor VIII were about 80% lower than those found in C57BL/6 mice (Fig 5C). As observed in rats, factor VIII activity was also significantly reduced in C57BL/6 mice injected with BjV, particularly at 6 h (p = 0.001) and 24 h (p = 0.05). Due to the low levels of factor VIII in *Vwf*^*-/-*^ mice, no statistically significant differences could be noticed between the saline and BjV groups over time.

Regardless of the presence of VWF in circulation, BjV induced the same proportion of thrombocytopenia in both strains over time (p = 0.072), and platelet counts in the BjV group was always lower than that of the respective controls (Fig 5D). Even though the extent of thrombocytopenia was similar in *Vwf*^*-/-*^ and C57BL/6 mice, MPV and the platelet distribution width (Fig 5E and 5F) in *Vwf*^*-/-*^ mice did not increase in the same extent as in C57BL/6 mice.

These findings support the reasoning that consumption/cleavage of VWF is not associated with thrombocytopenia during *B*. *jararaca* envenomation, and that botrocetin is not an important toxin involved in the development of thrombocytopenia during envenomation. Thus, the presence of VWF may be a minor contributing factor, but not a determinant factor, for BjV-induced thrombocytopenia.

## Discussion

Pathophysiological events that occur during snakebite envenomation are complex, and are a consequence of multiple toxins acting in concert to disturb homeostasis. This investigation complements previous studies to understand the function and participation of BjV toxins that act on hemostasis, particularly those that trigger thrombocytopenia.

In previous articles, the catalytic activity of SVMP and SVSP and intravascular thrombin generation have been shown not to be involved in the development of thrombocytopenia in models of *B*. *jararaca* envenomation [56,57]. Once botrocetin is one the best known CTLP in BjV, whose *in vitro* and *in vivo* mechanisms of action have been scrutinized, our leading hypothesis was that botrocetin, would be involved in the development of thrombocytopenia. In fact, aspercetin, a CTLP from *Bothrops asper* snake venom quite similar to 2-chain botrocetin, had been shown to be the main toxin in the development of thrombocytopenia induced by *B*. *asper* administration [66,67]. By incubating anti-aspercetin polyclonal antibodies with *B*. *asper* venom, using an experimental model similar to the one we used herein for rats, Rucavado et al. were able to demonstrate that aspercetin was the main toxin involved in the thrombocytopenia induced by *B*. *asper* envenomation [66]. However, the same anti-aspercetin antibody, when preincubated with BjV, could not prevent the development of BjV-induced thrombocytopenia. Thus, CTLP similar to either botrocetin or aspercetin were not involved in the development of thrombocytopenia during *B*. *jararaca* envenomation. Purified CTLP that directly bind to GPIb from *Bothrops caribbaeus* venom [68] and BjV (GPIb-BP, [42]), without the participation of VWF, have been shown to induce thrombocytopenia when injected in animals, but experimental evidence must still be obtained showing they are important toxins to induce thrombocytopenia during *B*. *jararaca* envenomation.

Once the experimental model of BjV preincubated with ABA showed that botrocetin was not involved in the development of thrombocytopenia, to double prove that botrocetin was not involved therein, mice that are deficient in VWF were also used. Once again, no reduction in the extent of thrombocytopenia was noticed, demonstrating that botrocetin is not the main toxin that causes platelet consumption during envenomation in rats and mice. A previous study also reported that purified botrocetin did not cause thrombocytopenia in models of VWD, depending thereby on the presence of circulating VWF [52]. Since intense thrombocytopenia was noticed even in *Vwf*^-/-^ mice, all these results stand as a proof that botrocetin is not the main toxin involved in thrombocytopenia during *B*. *jararaca* envenomation.

Although botrocetin showed no relevant role in the development of thrombocytopenia, the findings shown herein indicate that it alters VWF levels and function. BjV administration elicited low VWF:Ag and VWF:CB levels, a drop in HMWM-VWF, and a fall in FVIII levels in rats. In C57BL/6 mice, there were normal or reduced VWF:Ag levels, and depletion of HMWM-VWF [47]. These types of alteration resemble the laboratorial manifestations of acquired VWD [69] or congenital type 2A VWD. The latter is characterized by normal or reduced VWF:Ag levels, low VWF-dependent platelet adhesion, CB/Ag ratio lower than ≤ 0.6 and selective deficiency of HMWM-VWF [70]. Therefore, our results demonstrate that during *B*. *jararaca* envenomation, rats and mice underwent VWF alterations induced by botrocetin and SVMP that mimic those observed in patients with type 2A VWD. Conveying these findings to patients bitten by *B*. *jararaca*, consumption and proteolysis of VWF by botrocetin and SVMP, respectively, seem to contribute to the manifestation of bleedings, particularly those from mucosae (gingival bleeding, hematuria, and epistaxis).

Thus, our findings support the view that HMWM-VWF is both consumed by botrocetin and also cleaved by SVMP during envenomation. Concomitantly, HMWM-VWF seem to be released by endothelial cells and platelets, in response the injury evoked by BjV toxins or inflammatory mediators. Apparently, a complex dynamic process occurs between synthesis, proteolysis (either by ADAMTS13 or SVMP) and clearance of the VWF from circulation. In fact, it was clear that even though many individuals had lost HMWM-VWF, they showed an increase in VWF:Ag levels. This likely meant that (a) in the ELISA used to quantify VWF:Ag was increasing the absorbance of samples whose VWF has been cleaved, because the coating antibodies would be binding to newly exposed epitopes in cleaved VWF molecules, amplifying thereby the final absorbance signal, (b) or that there was a simultaneous stimulation of endothelial cells to release HMWM during envenomation, which would increase VWF:Ag levels. As these two hypotheses could be occurring simultaneously, we tried to assay VWF propeptide [71] to test whether HMWM-VWF secretion by endothelial cells or platelets was enhanced during envenomation, but we failed to find any commercially available kit specific for the detection of rat or mouse VWF propeptide.

Another interesting result was the mild reduction of ADAMTS13 activity in rats, accompanied by a reduction in VWF levels and cleavage of HMWM-VWF. The latter ones are intrinsically linked physiologically to augmented ADAMTS13 activity. Our results showed that Na_2_-EDTA could block the aforementioned observations, demonstrating that SVMP are linked to them, i.e, altering ADAMTS13 function and proteolyzing VWF. In addition, ADAMTS13 has already been shown to be cleaved by thrombin and plasmin [72], two enzymes profusely generated in circulation during *B*. *jararaca* envenomation [5,14], entailing thereby the decreased ADAMTS13 activity. Interestingly, prior to the discovery of the association between severe ADAMTS13 deficiency and thrombotic thrombocytopenic purpura (TTP), intravenous administration of purified botrocetin has been claimed to mimic the laboratorial picture of acute TTP: thrombocytopenia, formation of platelet microthrombi in lungs and spleen, and depletion of circulating VWF, particularly of HMWM-VWF [52,53]. Although rarely diagnosed, some patients bitten by *B*. *jararaca* also manifest thrombotic microangiopathy [73,74], but their ADAMTS13 levels were normal as well as in others bitten by other snakes [75], indicating that venom toxins are directly involved in the phenomena of activation of coagulation, endothelial injury, and red blood cell lysis and poikilocytosis. Paradoxically, mice that received BjV showed a decrease in *Adamts13* mRNA synthesis in the liver [65]. Since ADAMTS13 is not only produced by the hepatic stellate cells in liver, but also by endothelial cells [76], gene expression of *Adamts13* still needs to be studied in other organs, to evaluate whether negative feedback occurs.

Levels of FVIII and fibrinogen were reduced in envenomed rats and mice, similarly as observed in humans envenomed by *B*. *jararaca* [5,14]. However, taking into consideration the extent of reduction in FVIII/VWF:Ag ratio, the drop in FVIII levels seems to be more intense than the fall in VWF:Ag levels, indicating that there was a direct consumption of FVIII, independent of VWF cleavage. Moreover, different from the consumption of fibrinogen, inhibition of SVMP apparently did not prevent FVIII consumption in rats. Factor V levels steadily dropped in envenomed animals, and was inhibited by SVMP inhibition, showing that meizothrombin or factor Xa, generated by prothrombin or factor X activators found in BjV, might be involved therein.

In conclusion, our results show that botrocetin is not the main toxin that induce thrombocytopenia, and that VWF is consumed and cleaved during envenomation by *B*. *jararaca* snakes, similarly to what happens in acquired or type 2A VWD. Concomitantly to the proteolysis of the VWF, endothelial cells and platelets, in response to the direct injury induced by BjV toxins or inflammatory mediators, seem to secrete new molecules of HMWM-VWF in circulation. Besides the alterations in VWF, FVIII is consumed during envenomation, not only as a consequence of VWF cleavage. Overall, this study shows the complexity of the pathophysiological events induced by BjV, and highlight that there are mechanisms still unknown and/or proteins in BjV involved in the rapid platelet consumption, and that the disturbances in VWF may contribute to the bleeding manifestations of patients bitten by *B*. *jararaca* snakes.

## Methods

### Ethics statement

Human donors: adult healthy volunteers, who did not report the use of any medication affecting hemostasis during the previous 10 days to blood collection, signed a written consent in accordance with the Declaration of Helsinki and national regulations. This study was approved by the National Human Research Ethics Committee (Plataforma Brasil, CAAE 37958514.8.0000.0086, Ministry of Health, Brazil).

Experimental animals: VWF knock-out mice (*Vwf*^-/-^) were obtained by breeding heterozygous B6.129S2-*Vwf*^*tm1Wgr*^/J [62] mice purchased from The Jackson Laboratory (stock #003795, JAX, USA). Isogenic controls (C57BL/6) were used as controls. All *Vwf*^-/-^ mice were genotyped according to the protocol suggested by The JAX Laboratory. One male rabbit, male Wistar rats (250–300 g), and male *Vwf*^-/-^ and C57BL/6 mice (25–28 g) were bred at the Animal Facility, Instituto Butantan. Animals had free access to water and food, and were bred and maintained in a standardized environment, in rooms with defined flow of people, materials and supplies. In addition, they were continuously protected by health status barriers (barrier autoclave, HEPA air filtration system, differential pressure, etc.). To control ammonia in the environment, the exhausting system was kept at 15 to 20 air changes/h at room level. The light cycle was defined as 12-h light: 12-h dark. All procedures involving the use of animals followed National Guidelines (Conselho Nacional de Controle de Experimentação Animal, CONCEA, Brazil), were in accordance with ARRIVE (Animals in Research: Reporting In Vivo Experiments) guidelines, and were approved by the Institutional Animal Care and Use Committee in Instituto Butantan (CEUAIB 5232120618, 5937060618, 8847060516 and 2181021015).

### Bothrops jararaca *venom*

Lyophilized crude venom from a pool of adult specimens of *Bothrops jararaca* snakes was obtained from the Laboratory of Herpetology, Instituto Butantan (Sistema Nacional de Gestão do Patrimônio Genético e do Conhecimento Tradicional Associado, SisGen AF375C2). It was maintained frozen at -20°C until the moment of use. *Bothrops* antivenin (SAB, lot 1305077) was kindly donated by Instituto Butantan.

### Botrocetin and anti-botrocetin antibodies (ABA)

Partially purified 2-chain botrocetin from BjV was kindly donated by Dr. Solange M.T. Serrano, Instituto Butantan. It was further purified to homogeneity and characterized, as described in the supporting information (Fig D in S1 Text). The sequences obtained from the purified botrocetin used herein showed higher identity to botrocetin-2, an isoform of botrocetin [41]. Polyclonal anti-botrocetin (ABA) antibodies were obtained by immunizing a rabbit with the purified protein, and detailed protocols for purification and characterization of ABA are also described in the supporting information. Purified botrocetin induced platelet aggregation in human and rat PRP, but not in washed platelets, and the preincubation of ABA with botrocetin inhibited botrocetin-induced aggregation in human and rat platelets (Fig E and F in S1 Text).

### Envenomation protocols

Two models were used for the *in vivo* experiments: (1) Rats injected with BjV preincubated with ABA and/or Na_2_-EDTA, and (2) *Vwf*^-/-^ and background control mice (C57BL/6), injected with BjV. In both models, rats and mice were randomly allocated in experimental groups (www.randomizer.com), BjV was injected s.c. at the dose of 1.6 mg/kg b.w [56,58], in the dorsal region of both species. At 3, 6 and 24 h after venom or vehicle injection, animals were anesthetized (4% isoflurane for induction and 2.5% for maintenance, under continuous oxygen flux at 0.5%, Harvard Apparatus), and blood was collected.

For rats, in the moment of use, crude BjV was diluted in sterile saline. (1) **Group BjV+saline**, i.e., the positive control group: 1 mL of BjV (1 mg/mL in saline) was incubated for 1 h at 37°C with saline (52 μL); (2) **Group BjV+ABA**, i.e., the group whose botrocetin in BjV was neutralized by antibodies: 0.5 mL of BjV (2 mg/mL) was incubated for 1 h at 37°C with 0.5 mL of ABA (1.125 g/dL) and saline (52 μL); (3) **Group BjV+Na**_**2**_**-EDTA**, i.e., the group whose SVMP in BjV were inhibited by Na_2_-EDTA: 1 mL of (1 mg/mL) was incubated for 1 h at 37°C with 269 mM Na_2_-EDTA (52 μL) [57]; (4) **Group BjV+ABA+Na**_**2**_**-EDTA**, i.e., the group whose both botrocetin and SVMP in BjV were inhibited: 0.5 mL of BjV (2 mg/mL) was incubated for 1 h at 37°C with 0.5 mL de ABA (1.125 g/dL) and 269 mM Na_2_-EDTA (52 μL); and (5) **Saline control group**, i.e., the negative control group: rats were injected with saline.

For mice: *Vwf*^-/-^ and controls were injected with BjV or vehicle (saline).

### Blood collection

Blood samples were collected from the abdominal aorta of both species, with the guidance of a stereo microscope M60 (Leica, USA). After collection, blood samples were immediately transferred to tubes containing or not anticoagulants, and kept at room temperature during their processing. SAB was also added to the tubes, in the proportion of 1:100 (v/v), for the *in vitro* neutralization of BjV activity on cells and proteins. Different anticoagulants were used: (a) 269 mM Na_2_-EDTA, in the proportion of 1/100, for complete blood cell counts (CBC); (b) CTAD [16], in the proportion of 1/7, for evaluation of VWF:Ag, VWF:CB and fibrinogen assay; (c) 3.2% trisodium citrate, in the proportion of 1:10, for factors VIII and V assays; (d) 3.8% trisodium citrate with inhibitors [77], in the proportion of 1/10, for electrophoresis of VWF multimers; (e) glass tubes with neither anticoagulant nor SAB were used for obtaining serum samples and assaying circulating venom levels (venenemia). Poor platelet plasma and serum were obtained by centrifugation at 2,500 *g* for 15 min at room temperature, and the samples were maintained at room temperature during the whole process. Plasma and serum aliquots were rapidly stored at −80°C.

### Assays

Venenemia was determined by ELISA in serum samples from mice and rats, as described previously [57]. Plasma fibrinogen was assayed according to Ratnoff and Menzie’s method [78]. CBC were determined in an automated veterinary cell counter (BC-2800 Vet, Mindray, China).

### VWF assays

VWF antigen (VWF:Ag) was assayed in all plasma samples, including in those of *Vwf*^-/-^ mice, as reported earlier [16]. Since rat VWF is not responsive to ristocetin [79,80], the VWF collagen binding (VWF:CB) assay was chosen for evaluating the functional activity of VWF, i.e., the interaction between VWF and the subendothelial-matrix, rather than the VWF ristocetin cofactor (VWF:RCo) assay, which evaluates the interaction between VWF and platelet GPIbα. Determination of rat VWF:CB followed a previous protocol [81], with modifications. Briefly, 96-well MaxiSorp (Nunc) microplates were sensitized with 100 μL/well of 0.25 mg/mL type III collagen from human placenta (code C4407, Sigma, USA) in 0.1 M carbonate buffer (pH 9.6) for 24 h at room temperature in a humid chamber. The microplates were then blocked with 200 μL/well of 3% bovine serum albumin (BSA) in carbonate buffer for 2 h at 37°C in a humid chamber. Samples were diluted 1/40 in incubation buffer (1% BSA, 0.05% Tween 20 in PBS pH 7.4), and aliquots of calibration standards (a plasma pool of control rats twofold serially diluted, from 1/10 to 1/20480) were then added to wells, and the plate was incubated at 37°C for 1 h in a humid chamber. After extensive washing, 100 μL/well of the primary antibody (rabbit anti-human VWF, code A0082, Dako, Denmark) diluted (1/1000) in incubation buffer was added, and the microplates were incubated at 37°C for 1 h in a humid chamber. Then, after extensive washing, 100 μL/well of the secondary antibody (peroxidase-conjugated goat anti-rabbit IgG, code A0545, Sigma, USA), diluted 1/1000 in incubation buffer, was added and microplates were incubated for 1 h at 37°C in a humid chamber. The reaction was developed and halted as reported elsewhere [82], and absorbance was read at 492 nm. The dilution of 1/40 was considered as 100% of VWF:CB activity. In order to verify the quality and reproducibility of VWF:Ag and VWF:CB determinations, a normal rat sample was considered as a reference and assayed in all microplates used.

Analysis of VWF multimer distribution was determined essentially as described previously [47], using as development the goat anti-rabbit IgG conjugated with Alexa Fluor 647 (Invitrogen, A21245, USA). For each sample, the optical density of HMWM-VWF was expressed as a relative percentage of the total optical density of the respective lane, and thus the percentage of HMWM-VWF could be compared between different samples. In all analyses, a sample of a normal plasma pool was analyzed and designated as the 100% reference for the HMWM. Therefore, all test samples were normalized in relation to the reference sample.

### Assay of ADAMTS13 activity

The commercial kit Actifluor ADAMTS13 Activity (Sekisui Diagnostics, EUA) was used to assay ADAMTS13 activity in rat plasma samples, according to the manufacturer’s instructions. The increase in fluorescence during 1 h was used to calculate ADAMTS13 activity.

### Assays of factors VIII and V

Levels of factors VIII and V in rat plasma samples were determined using the respective commercial deficient plasmas (Diagnostica Stago, France). Plasma samples were diluted 1/10 (considered as 100%) in Tyrode buffer without calcium, and the assay was performed according to the manufacturer’s instructions. Clotting times were measured on a Start4 coagulometer (Stago, France).

### Ratios

The ratios (a) *CB/Ag*, i.e., the ratio of VWF:CB to VWF:Ag, and (b) *FVIII/VWF*:*Ag*, i.e., the ratio of the FVIII coagulant activity to VWF:Ag were also calculated. *CB/Ag* was used to analyze the proportion of alteration in the biological function of VWF in comparison to antigen levels. The FVIII/VWF:Ag ratio was obtained by dividing the value of the FVIII coagulant activity by the respective VWF:Ag value, assisting thereby in the analysis of the proportion of FVIII bound to VWF.

### Statistical analyses

The presence of outliers was initially detected as described elsewhere [83], using a Microsoft Excel 2013 spreadsheet for calculations. Thereafter, the data were analyzed for normal distribution and homoscedasticity in Stata statistical software (version 10.0), and, whenever necessary, data were transformed to obtain homoscedasticity. Two-way ANOVA, followed by Sidak’s test, was used to compare data from rats in SPSS (version 22). Data that showed neither homoscedasticity nor normality, even after transformation, were analyzed using Aligned Rank Transform [84] in R software (version 4.0.0). For the analysis of correlation between the HMWM-VWF and VWF:CB, Pearson’s correlation coefficient (r) was used; confidence intervals (95%) for the correlation value was estimated using a percentile bootstrap, using 1000 replicates, in SPSS (version 22). For analyses of mouse data, the strain was also taken into account in the statistical analyses, and three-way ANOVA, followed by Sidak’s test, was performed in SPSS (version 22). Values of p <0.05 were considered statistically significant. Data were presented as mean ± standard error of mean (s.e.m.).

## Supporting information

S1 Text **Fig A**. Venenemia in rats 3, 6 and 24 h after BjV administration. Rats were injected with saline alone (saline control), or BjV previously incubated with saline (BjV+saline), anti-botrocetin antibodies (ABA), BjV+Na2-EDTA, or BjV+ABA+Na2-EDTA. Venom levels in serum were assayed by ELISA. ① Statistically significant different (p <0.05) from the saline control on the respective time. Data are expressed as mean ± s.e.m (n = 6–16 rats/group per time period). **Fig B**. Scatterplot and linear regression of paired observations between VWF:CB and HMWF-VWF in rats at 3, 6 and 24 h after s.c. injection of saline (Saline Control) or BjV+saline. Pearson’s correlation was used, and results were expressed as correlation coefficient (r), and the 95% confidence interval are shown between brackets. **Fig C**. Levels of circulating venom levels (**a**), red blood cell counts (RBC, **b**), hemoglobin (**c**), hematocrit (**d**), and white blood cell counts (WBC, **e**) in Vwf-/- and C57BL/6 mice at 3, 6 and 24 h after injection of saline alone or BjV. ① Group statistically different (p <0.05) from the saline control group at the respective time. ②- The response of Vwf-/- mice was statistically different (p <0.05) from that of C57BL/6 mice at the respective time and treatment. Data are expressed as mean ± s.e.m (n = 5–7 mice/group/time period). **Fig D**. (a) SDS-PAGE of 2-chain botrocetin purified from BjV, under non-reduction and reduction conditions. Proteins were silver-stained. The image does not reflect the cropping from different gels. (b) The protein bands observed under reducing conditions were sliced from the gel and submitted to in-gel trypsin digestion, and mass spectrometric analysis by LC–MS/MS. **Fig E**. Western blotting of BjV and 2-chain botrocetin. BjV (10 μg) and 2-chain botrocetin (1 μg) were electrophoresed in 12% SDS-PAGE, and transferred onto a nitrocellulose membrane. The membrane was incubated with ABA (dilution 1/10,000), followed by incubation with 1/5,000 anti-rabbit IgG conjugated with peroxidase (Sigma A0545) and development. This image does not reflect the cropping from different gels or blottings. **Fig F**. Platelet aggregation induced by botrocetin and its inhibition by ABA. Human or rat PRP (400 μL, 300 × 109/L) was stimulated by 10 μL of botrocetin (final concentration, 2.4 μg/mL) previously incubated with ABA or vehicle (50% glycerol). Tracings are representative of three different experiments.
(DOCX)
