# Supplementary material for: Involvement of von Willebrand factor and botrocetin in the thrombocytopenia induced by Bothrops jararaca snake venom
Source: PLoS Negl Trop Dis. 2021 Sep 3;15(9):e0009715. doi: 10.1371/journal.pntd.0009715 (PMC8445451; doi:10.1371/journal.pntd.0009715)
Supplement: S1 Text — Fig A. Venenemia in rats 3, 6 and 24 h after BjV administration. Rats were injected with saline alone (saline control), or BjV previously incubated with saline (BjV+saline), anti-botrocetin antibodies (ABA), BjV+Na2-EDTA, or BjV+ABA+Na2-EDTA. Venom levels in serum were assayed by ELISA. ① Statistically significant different (p <0.05) from the saline control on the respective time. Data are expressed as mean ± s.e.m (n = 6–16 rats/group per time period). Fig B. Scatterplot and linear regression of paired observations between VWF:CB and HMWF-VWF in rats at 3, 6 and 24 h after s.c. injection of saline (Saline Control) or BjV+saline. Pearson’s correlation was used, and results were expressed as correlation coefficient (r), and the 95% confidence interval are shown between brackets. Fig C. Levels of circulating venom levels (a), red blood cell counts (RBC, b), hemoglobin (c), hematocrit (d), and white blood cell counts (WBC, e) in Vwf-/- and C57BL/6 mice at 3, 6 and 24 h after injection of saline alone or BjV. ① Group statistically different (p <0.05) from the saline control group at the respective time. ②- The response of Vwf-/- mice was statistically different (p <0.05) from that of C57BL/6 mice at the respective time and treatment. Data are expressed as mean ± s.e.m (n = 5–7 mice/group/time period). Fig D. (a) SDS-PAGE of 2-chain botrocetin purified from BjV, under non-reduction and reduction conditions. Proteins were silver-stained. The image does not reflect the cropping from different gels. (b) The protein bands observed under reducing conditions were sliced from the gel and submitted to in-gel trypsin digestion, and mass spectrometric analysis by LC–MS/MS. Fig E. Western blotting of BjV and 2-chain botrocetin. BjV (10 μg) and 2-chain botrocetin (1 μg) were electrophoresed in 12% SDS-PAGE, and transferred onto a nitrocellulose membrane. The membrane was incubated with ABA (dilution 1/10,000), followed by incubation with 1/5,000 anti-rabbit IgG conjugated with perox [file pntd.0009715.s001.docx]

**Involvement of von Willebrand factor and botrocetin in the thrombocytopenia induced by *Bothrops jararaca* snake venom**

Camila Martos Thomazini^1,6^, Ana Teresa Azevedo Sachetto^1,6^, Cynthia Zaccanini de Albuquerque^2^, Vânia Gomes de Moura Mattaraia^2^, Ana Karina de Oliveira^3^, Solange Maria de Toledo Serrano^3^; Ivo Lebrun^4^, Katia Cristina Barbaro^5^, Marcelo Larami Santoro^1,6,^*

^1^Laboratório de Fisiopatologia; ^2^Biotério Central; ^3^Laboratório de Toxinologia Aplicada, Center of Toxins, Immune-Response and Cell Signaling (CeTICS); ^4^Laboratório de Bioquímica e Biofísica; ^5^Laboratório de Imunopatologia, Instituto Butantan, São Paulo-SP. ^5^Programa de Pós-Graduação em Clínica Médica, Faculdade de Medicina, Universidade de São Paulo, Brazil.

*Correspondence should be addressed to:

Marcelo L. Santoro, DVM, MSc, PhD

Instituto Butantan

Lab. de Fisiopatologia

Av. Dr. Vital Brasil, 1500

05503-900 São Paulo-SP

Tel. +55-11-2627-9743

E-mail: marcelo.santoro@butantan.gov.br / marcelosantoro@yahoo.com

**Supporting information**





**Fig A.** Venenemia in rats 3, 6 and 24 h after BjV administration. Rats were injected with saline alone (saline control), or BjV previously incubated with saline (BjV+saline), anti-botrocetin antibodies (ABA), BjV+Na_2_-EDTA, or BjV+ABA+Na_2_-EDTA. Venom levels in serum were assayed by ELISA. ① Statistically significant different (p <0.05) from the saline control on the respective time. Data are expressed as mean ± s.e.m (n = 6-16 rats/group per time period).


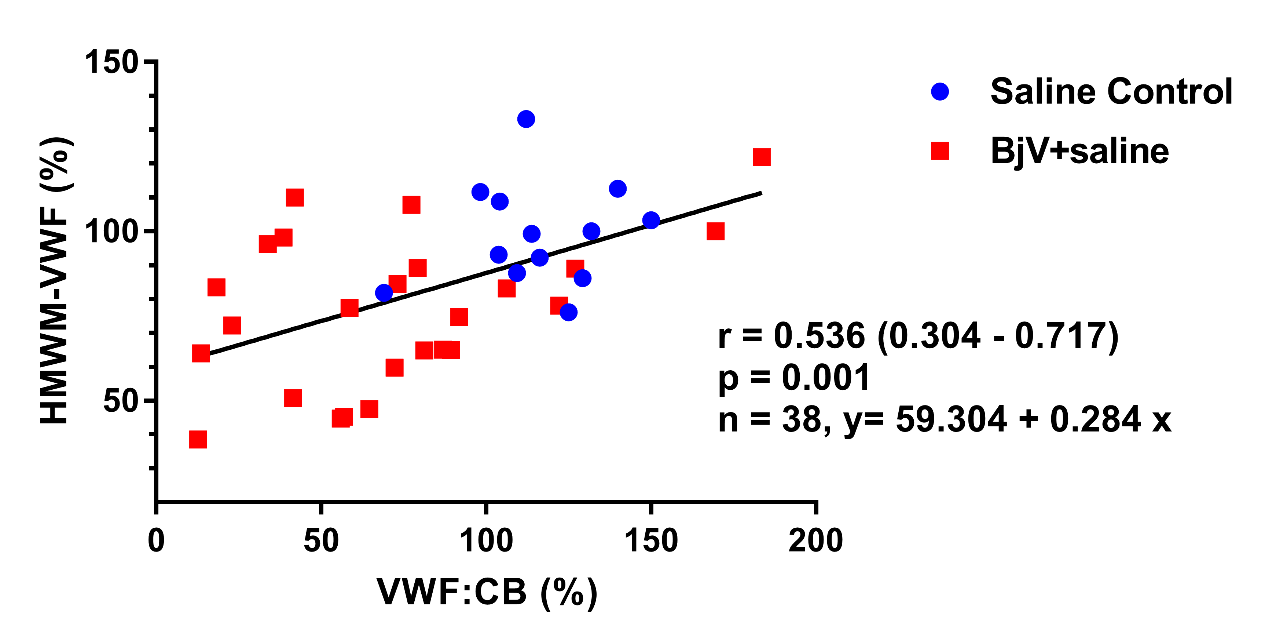


**Fig B.** Scatterplot and linear regression of paired observations between VWF:CB and HMWF-VWF in rats at 3, 6 and 24 h after s.c. injection of saline (Saline Control) or BjV+saline. Pearson’s correlation was used, and results were expressed as correlation coefficient (r), and the 95% confidence interval are shown between brackets.


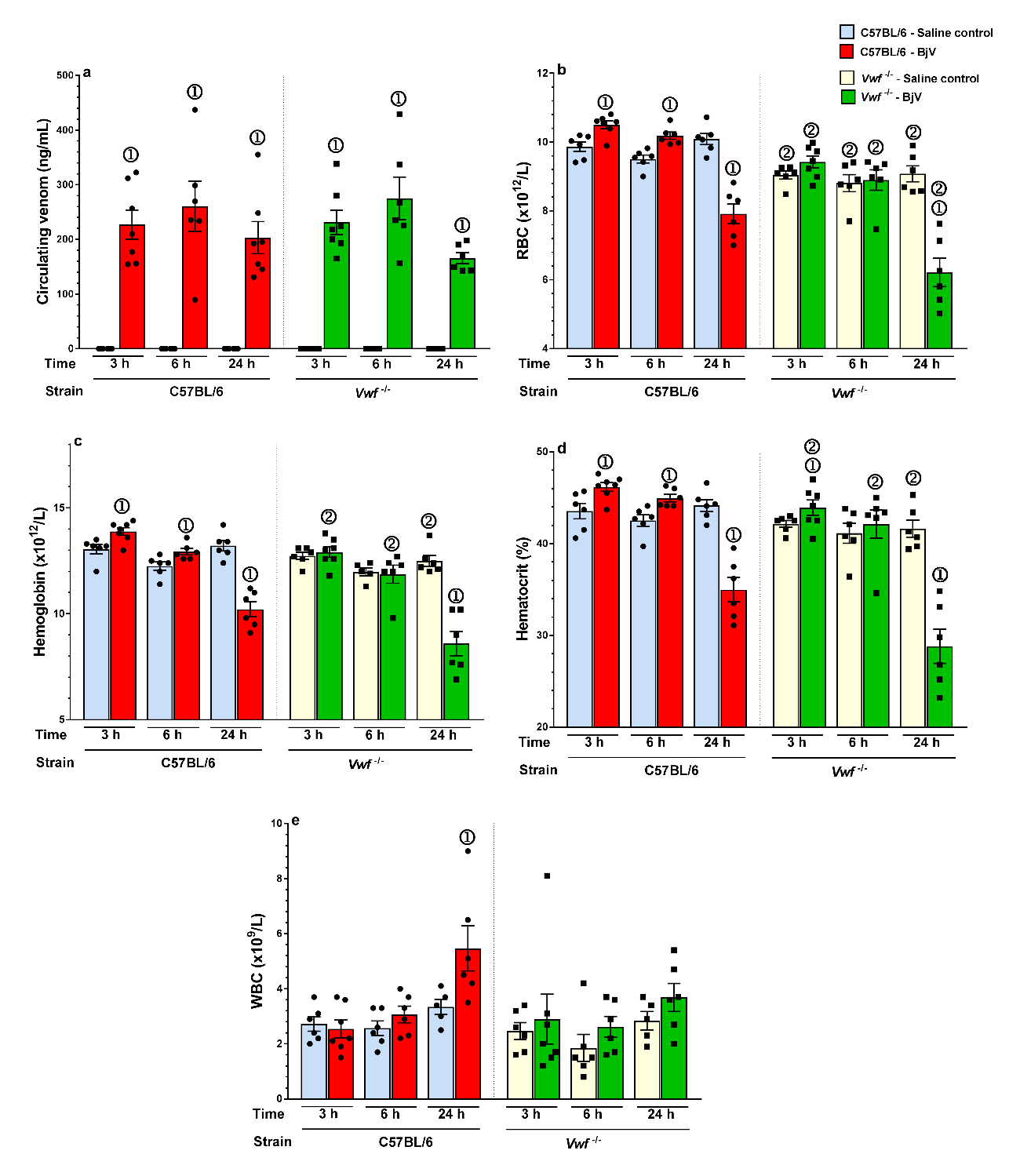


**Fig C**. Levels of circulating venom levels (**a**), red blood cell counts (RBC, **b**), hemoglobin (**c**), hematocrit (**d**), and white blood cell counts (WBC, **e**) in *Vwf^-/-^* and C57BL/6 mice at 3, 6 and 24 h after injection of saline alone or BjV. ① Group statistically different (p <0.05) from the saline control group at the respective time. ②- The response of *Vwf*^-/-^ mice was statistically different (p <0.05) from that of C57BL/6 mice at the respective time and treatment. Data are expressed as mean ± s.e.m (n = 5-7 mice/group/time period).

*Purification of 2-chain botrocetin from BjV and production of polyclonal anti-botrocetin antibodies* (ABA)

Partially purified botrocetin, a by-product of purification of a phospholipase A_2_ from BjV [1], was kindly donated by Dr. Solange M.T. Serrano, Instituto Butantan. Analyses by LC-MS/MS revealed that the botrocetin fraction was contaminated with an acid phospholipase A_2_ and SVMP, and then it was further subjected to chromatographic separation in a C8 column in a HPLC system [2]. After analysis by SDS-PAGE, the protein peak that had molecular masses equivalent to the two subunits of botrocetin was concentrated (0.39 mg protein/mL) and analyzed by SDS-PAGE. Non-reduced 2-chain botrocetin had a relative molecular mass of 22-23 kDa, and subunit α and β had molecular masses of 16 and 14.5 kDa, respectively (Fig D). The non-reduced band was excised from the gel, reduced, alkylated, trypsin digested, and analyzed by LC/MS/MS (Fig D), and showed that the botrocetin used herein showed high identity to botrocetin-2 [3].


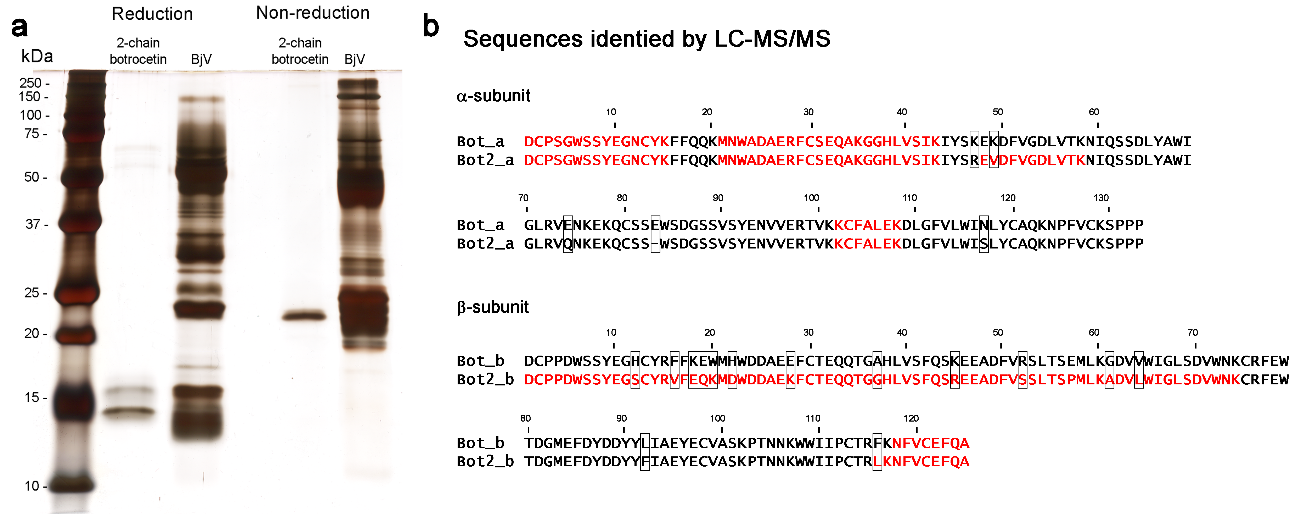


**Fig D.** (a) SDS-PAGE of 2-chain botrocetin purified from BjV, under non-reduction and reduction conditions. Proteins were silver-stained [4]. The image does not reflect the cropping from different gels. (b) The protein bands observed under reducing conditions were sliced from the gel and submitted to in-gel trypsin digestion [5], and mass spectrometric analysis by LC–MS/MS. The resulting peptide mixture was dried and dissolved in 20 μL of 0.1% formic acid (solution A). The peptide mixture (5 μL) was injected into a 2 cm C-18 trap column (100 μm I.D. × 360 μm O.D.; EASY II-nanoLC system) coupled to an LTQ-Orbitrap Velos mass spectrometer (Thermo Fisher Scientific). Chromatographic separation of tryptic peptides was performed on 10 cm long column (75 μm I.D. x 360 μm O.D.) packed with 5 μm Aqua C-18 beads (Phenomenex). Peptides were eluted with a linear gradient of 5−95% acetonitrile in 0.1% formic acid (solution B) at 200 nL/min: 5−95% in 15 min, remaining at this concentration for 5 min, back to 5% in 1 min and 5%B for 19 min. Spray voltage was set at 2.0 kV and the mass spectrometer was operated in data dependent mode, in which one full MS scan was acquired in the m/z range of 200−2000 followed by MS/MS acquisition using collision induced dissociation of the five most intense ions from the MS scan. MS spectra were acquired in the Orbitrap analyzer at 30,000 resolution (at 400 m/z) whereas the MS/MS scans were acquired in the linear ion trap. Isolation window, activation time and normalized collision energy were set to, respectively, 2 m/z, 10 ms and 35%. A dynamic peak exclusion was applied to avoid the same m/z of being selected for the next 30 s. MS/MS raw data were converted to MGF format using the MS convert tool (ProteoWizard version 3.0.3535, http://proteowizard.sourceforge.net/) for database searching using the Mascot server (version 2.2; Matrix Science, UK) against a target database restricted to the taxonomy “Serpentes” (UniProt released 2017; 70166 sequences, appended with 79 sequences derived from the translation of cDNAs encoding toxins in the *B. jararaca* venom gland [6, 7], with a parent tolerance of 10 ppm and fragment tolerance of 0.5 Da. Iodoacetamide derivatives of cysteine and oxidation of methionine were specified in Mascot, respectively, as fixed and variable modifications. The peptides identified in the analysis are marked in red, and showed high identity to botrocetin-2. Amino acid sequences of botrocetin subunit α (Bot_a, UniProtKB accession code P22029) and subunit β (Bot_b, UniProtKB accession code P22030) were aligned to botrocetin-2 sequences (Bot2_a and Bot2_b; UniProtKB accession codes M1V359 and M1VNP5, respectively) using ClustawW. The numbers indicate the distance to the beginning of botrocetin chains sequence. Differences in amino acid residues between botrocetin and botrocetin-2 are boxed in black.

*Anti-botrocetin antibodies (ABA)*

Purified 2-chain botrocetin was emulsified with Marcol-Montanide adjuvant, in the proportion of 1:2, and inoculated i.m. in an adult New Zealand rabbit. It received 4 additional boosters, and reached a titer greater than 204,800 by ELISA in a plate sensitized with 1 µg/well of BjV. IgG was purified from rabbit serum [8], incubated in a dry bath at 52°C for 20 min, and centrifuged at 3220 *g* for 20 min at 4°C. One volume of the supernatant containing the IgG was diluted with one volume of 100% glycerol and kept at - 20°C. The total protein concentration of this anti-botrocetin antibody solution was 1.125 g/dL [9].

*ABA reactivity and neutralization of botrocetin-induced platelet agglutination*

BjV proteins identified by ABA were analyzed by Western blotting (Fig E). Interestingly, under reducing conditions, only the band equivalent to the β chain was identified by ABA (14.5 kDa), evidencing that the α alpha chain was less immunogenic. Under non-reducing conditions, ABA bound intensely to a band with molecular mass corresponding to non-reduced 2-chain botrocetin (21.9 kDa), but also to bands with 43 kDa and others showing higher molecular masses (> 150 kDa), suggesting that 2-chain botrocetin formed oligomers or was connected to SVMP [10].


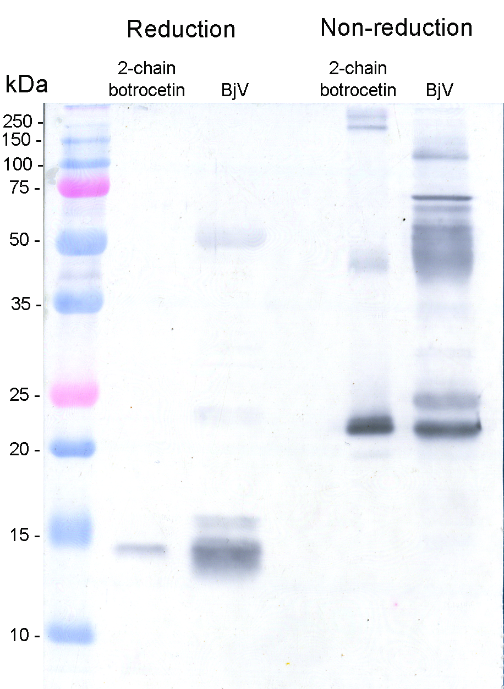


**Fig E**. Western blotting of BjV and 2-chain botrocetin. BjV (10 µg) and 2-chain botrocetin (1 µg) were electrophoresed in 12% SDS-PAGE, and transferred onto a nitrocellulose membrane. The membrane was incubated with ABA (dilution 1/10,000), followed by incubation with 1/5,000 anti-rabbit IgG conjugated with peroxidase (Sigma A0545) and development [11]. This image does not reflect the cropping from different gels or blottings.

Neutralization of botrocetin-induced platelet aggregation of human and rat platelets by ABA was evaluated. Blood samples from rats and humans were collected in 3.8% trisodium citrate, and platelet-rich plasma (PRP) was obtained by centrifugation at 190 *g* for 20 min at 25°C. Platelet-poor plasma (PPP) was obtained by centrifugation of blood samples at 2500 *g* for 15 min at 25°C, and diluted to reach platelet counts of 300 × 10^9^/L. One volume of purified 2-chain botrocetin (0.39 mg/mL) was incubated with 3 volumes of ABA or vehicle (50% glycerol) for 30 min at 37°C. For the platelet aggregation assays, 400 µL of PRP was incubated for 1 min in a platelet aggregometer (Chrono-log, model 560VS, USA) at 37°C, and then with 10 µL of this mixture (botrocetin previously incubated with ABA or vehicle) was added. As it can be observed in Fig F, ABA was efficient to inhibit platelet aggregation induced by botrocetin, in both humans and rats, demonstrating that the antibody was effective to neutralize the biological activity of botrocetin.





**Fig F**. Platelet aggregation induced by botrocetin and its inhibition by ABA. Human or rat PRP (400 µL, 300 × 10^9^/L) was stimulated by 10 µL of botrocetin (final concentration, 2.4 µg/mL) previously incubated with ABA or vehicle (50% glycerol). Tracings are representative of three different experiments.

Inhibition of the catalytic activities of SVMP and SVSP by ABA was also tested, taking into account the proportion of BjV and ABA used *in vivo* experiments. The hydrolysis of the chromogenic substrate for SVSP, benzoyl-DL-arginyl-p-nitroanilide (BAPNA), was evaluated as described earlier [12]; mean absorbance readings at 405 nm for BjV incubated with ABA (1.41, n= 3) or vehicle (1.40, n = 3 ) was not different, showing that SVSP were not being inhibited by ABA. Similarly, the coagulant activity of BjV on rat plasma, which is almost completely dependent on SVMP [13], was also evaluated [12] in the presence or absence of ABA. BjV preincubated with saline had a mean coagulant dose (MCD) of 13.28 µg/mL, whereas in the presence of ABA it was 12.55 µg/mL. As a positive control of the inhibition of the coagulant activity, BjV preincubated with 13 mM Na_2_-EDTA [13], a SVMP inhibitor, had an MCD of 352.74 µg/mL. These results demonstrated that ABA did not inhibit the catalytic activity of SVMP nor SVSP.

**References**

1. Serrano SMT, Reichl AP, Mentele R, Auerswald EA, Santoro ML, Sampaio CAM, et al. A novel phospholipase A_2_, BJ-PLA_2_, from the venom of the snake *Bothrops jararaca*: purification, primary structure analysis, and its characterization as a platelet-aggregation-inhibiting factor. Archives of Biochemistry & Biophysics. 1999; 367: 26-32.

2. Fujimura Y, Titani K, Usami Y, Suzuki M, Oyama R, Matsui T, et al. Isolation and chemical characterization of two structurally and functionally distinct forms of botrocetin, the platelet coagglutinin isolated from the venom of *Bothrops jararaca*. Biochemistry. 1991; 30: 1957-64.

3. Yamamoto-Suzuki Y, Sakurai Y, Fujimura Y, Matsumoto M, Hamako J, Kokubo T, et al. Identification and recombinant analysis of botrocetin-2, a snake venom cofactor for von Willebrand factor-induced platelet agglutination. Biochemistry. 2012; 51: 5329-38.

4. Blum H, Beier H, Gross HJ. Improved silver staining of plant proteins, RNA and DNA in polyacrylamide gels. Electrophoresis. 1987; 8: 93-9.

5. Hanna SL, Sherman NE, Kinter MT, Goldberg JB. Comparison of proteins expressed by *Pseudomonas aeruginosa* strains representing initial and chronic isolates from a cystic fibrosis patient: an analysis by 2-D gel electrophoresis and capillary column liquid chromatography-tandem mass spectrometry. Microbiology. 2000; 146: 2495-508.

6. Junqueira-de-Azevedo IL, Bastos CM, Ho PL, Luna MS, Yamanouye N, Casewell NR. Venom-related transcripts from *Bothrops jararaca* tissues provide novel molecular insights into the production and evolution of snake venom. Molecular Biology & Evolution. 2015; 32: 754-66.

7. Andrade-Silva D, Zelanis A, Kitano ES, Junqueira-de-Azevedo IL, Reis MS, Lopes AS, et al. Proteomic and glycoproteomic profilings reveal that post-translational modifications of toxins contribute to venom phenotype in snakes. J Proteome Res. 2016; 15: 2658-75.

8. McKinney MM, Parkinson A. A simple, non-chromatographic procedure to purify immunoglobulins from serum and ascites fluid. J Immunol Methods. 1987; 96: 271-8.

9. Redinbaugh MG, Turley RB. Adaptation of the bicinchoninic acid protein assay for use with microtiter plates and sucrose gradient fractions. Analytical Biochemistry. 1986; 153: 267-71.

10. Eble JA. Structurally robust and functionally highly versatile - C-type lectin (-related) proteins in snake venoms. Toxins (Basel). 2019; 11.

11. Pukac LA, Carter JE, Morrison KS, Karnovsky MJ. Enhancement of diaminobenzidine colorimetric signal in immunoblotting. Biotechniques. 1997; 23: 385-8.

12. Antunes TC, Yamashita KM, Barbaro KC, Saiki M, Santoro ML. Comparative analysis of newborn and adult *Bothrops jararaca* snake venoms. Toxicon. 2010; 56: 1443-58.

13. Yamashita KM, Alves AF, Barbaro KC, Santoro ML. *Bothrops jararaca* venom metalloproteinases are essential for coagulopathy and increase plasma tissue factor levels during envenomation. PLoS Negl Trop Dis. 2014; 8: e2814.
